# Particulate Matter (PM_2.5_) from Biomass Combustion Induces an Anti-Oxidative Response and Cancer Drug Resistance in Human Bronchial Epithelial BEAS-2B Cells

**DOI:** 10.3390/ijerph17218193

**Published:** 2020-11-06

**Authors:** Regina Merk, Katharina Heßelbach, Anastasiya Osipova, Désirée Popadić, Wolfgang Schmidt-Heck, Gwang-Jin Kim, Stefan Günther, Alfonso García Piñeres, Irmgard Merfort, Matjaz Humar

**Affiliations:** 1 Department of Pharmaceutical Biology and Biotechnology, Institute of Pharmaceutical Sciences, Albert Ludwigs University Freiburg, 79104 Freiburg, Germany; merk.regina@gmx.de (R.M.); katharina.hesselbach@hotmail.de (K.H.); tulip_88@list.ru (A.O.); desiree.popadic@pharmazie.uni-freiburg.de (D.P.); 2 Department of Systems Biology and Bioinformatics, Leibniz Institute for Natural Product Research and Infection Biology-Hans-Knöll Institute (HKI), 07745 Jena, Germany; wolfgang.schmidt-heck@leibniz-hki.de; 3 Department of Pharmaceutical Bioinformatics, Institute of Pharmaceutical Sciences, Albert-Ludwigs University Freiburg, 79104 Freiburg, Germany; gwang-jin.kim@pharmakol.uni-freiburg.de (G.-J.K.); stefan.guenther@pharmazie.uni-freiburg.de (S.G.); 4 Centro de Investigación en Biología Celular y Molecular (CIBCM), Universidad de Costa Rica, 11501-2060 San José, Costa Rica; alfonso.garciapineres@ucr.ac.cr; 5 Escuela de Química, Universidad de Costa Rica, 11501-2060 San José, Costa Rica; 6 Spemann Graduate School of Biology and Medicine (SGBM), Albert-Ludwigs University Freiburg, 79104 Freiburg, Germany

**Keywords:** particulate matter from biomass combustion, Nrf2, anti-oxidative response, glutathione, cancer drug resistance

## Abstract

Nearly half of the world’s population relies on combustion of solid biofuels to cover fundamental energy demands. Epidemiologic data demonstrate that particularly long-term emissions adversely affect human health. However, pathological molecular mechanisms are insufficiently characterized. Here we demonstrate that long-term exposure to fine particulate matter (PM_2.5_) from biomass combustion had no impact on cellular viability and proliferation but increased intracellular reactive oxygen species (ROS) levels in bronchial epithelial BEAS-2B cells. Exposure to PM_2.5_ induced the nuclear factor erythroid 2-related factor 2 (Nrf2) and mediated an anti-oxidative response, including enhanced levels of intracellular glutathione (GSH) and nuclear accumulation of heme oxygenase-1 (HO-1). Activation of Nrf2 was promoted by the c-Jun N-terminal kinase JNK1/2, but not p38 or Akt, which were also induced by PM_2.5_. Furthermore, cells exposed to PM_2.5_ acquired chemoresistance to doxorubicin, which was associated with inhibition of apoptosis and elevated levels of GSH in these cells. Our findings propose that exposure to PM_2.5_ induces molecular defense mechanisms, which prevent cellular damage and may thus explain the initially relative rare complications associated with PM_2.5_. However, consistent induction of pro-survival pathways may also promote the progression of diseases. Environmental conditions inducing anti-oxidative responses may have the potential to promote a chemoresistant cellular phenotype.

## 1. Introduction

Globally, an estimated nine million premature deaths are linked to environmental pollution, which is about as many deaths as from cancer [1,2]. Nearly half of these deaths are attributed to fine particles released during indoor biomass fuel emissions [3,4]. Although a high proportion of the global population relies on using biomass for its energy demands, the consequences of exposure to ambient particles from biomass combustion for human health have only been poorly investigated [4]. In developed countries residential biomass combustion is also a major source of particle emission, gaining in popularity as a renewable CO_2_ neutral energy source [5,6]. Emissions from industrial or traffic-related sources are essentially under legislation, but residential combustion of biomass fuels, agricultural burning, and forest fires are largely uncontrolled and thus may result in particle concentration up to 3 mg/m^3^ or even higher [6,7]. Particles less than 2.5 µm in diameter (PM_2.5_) are considered to be particularly harmful, as they efficiently evade the mucociliary defense system and accumulate in the lung parenchyma [8,9].

Epidemiologic data suggest that exposure to biomass combustion is associated with cardiovascular or pulmonary diseases, including stroke, chronic obstructive pulmonary disease (COPD), pulmonary infections, asthma, and lung cancer [3,4,10]. Molecular investigations using biofuel combustion particles are mainly focused on toxicity and inflammation [11,12,13]. Previously we investigated the methylome and gene expression profile of PM_2.5_-exposed BEAS-2B bronchial epithelial cells, revealing multiple putative pathways for cancer development and progression, and indicating susceptibility to microbial infections or other pulmonary associated diseases [14,15]. We also observed the induction of a stress response that preserved cellular integrity during long-term exposure to PM_2.5_ and involved the mitogen activated protein kinase MAPK p38 [16].

Traffic-related and industrial particle emissions have been shown to induce oxidative stress, inflammation, and cell death [8,17,18,19]. However, cells have developed cytoprotective mechanisms to prevent cell damage. According to the hierarchical stress model, transcriptional activation of antioxidant genes prevents oxidative cell damage at low stress levels. If these enzymes fail to neutralize the effects of reactive oxygen species (ROS), escalation of oxidative stress can lead to inflammation and finally to the initiation of cell death [20,21]. Nrf2 has been described as an ubiquitous transcriptional regulator of phase II detoxification and antioxidant genes that are essential mediators of anti-oxidative and anti-inflammatory defense mechanisms [22,23]. Heme oxygenase-1 (HO-1) encoded by the *HMOX1* gene is a significant anti-oxidative enzyme, which plays a crucial role in maintaining the cellular redox homeostasis against oxidative stress [24]. Importantly, *HMOX1* is regulated by Nrf2 [25]. Reduced glutathione (GSH) is another key antioxidant in mammals, involved in reducing reactive species and inhibiting cell apoptosis [26,27]. Bioavailability of GSH is at least in part controlled by Nrf2 via transcriptional regulation of the gene for the catalytic subunit of the glutamate cysteine ligase (*GCLC*), the rate-limiting enzyme of GSH synthesis [26,28]. Additionally, cell death can be prevented by other mechanism, e.g., by Akt, which suppresses cell death by directly targeting pro- and anti-apoptotic proteins or by modulating the transcriptional response to apoptotic stimuli [29]. As most of these pathways are associated with ROS generation, inflammation or cellular survival, they are also closely connected to cancer initiation and progression [30].

Deregulation of cytoprotective pathways can result in acquired drug resistance. Cancer drug resistance, which can be generated by drug inactivation, drug efflux, target inactivation, inhibition of cell death or activation of alternative signaling pathways, is a severe problem and responsible for most relapses in cancer patients after successful initial therapy [31]. Signaling pathways, such as the PI3K/Akt and Nrf2, are reported to be involved in cancer drug resistance [29,32,33]. Recently, it has been reported that cigarette smoke promotes cancer drug resistance, probably via Akt-mediated regulation of efflux pumps [34]. However, it is unclear whether environmental pollution also influences cancer therapy by inducing cancer drug resistance. We previously observed PM_2.5_ particle internalization and accumulation in vesicular compartments in human bronchial epithelial BEAS-2B cells by transmission electron microscopy and the induction of a stress response that preserved cellular viability during long-term exposure to PM_2.5_ from biomass combustion and involved MAPK p38, heat shock protein (HSP)-27, AMPK, and autophagy [16]. In continuation of our studies we analyzed the anti-oxidative response of human bronchial epithelial BEAS-2B cells upon long-term exposure to PM_2.5_ from biomass combustion and its potential role in the induction of drug resistance.

## 2. Materials and Methods

### 2.1. Ambient Particulate Matter (PM_2.5_)

The collection, characterization, and intracellular distribution of PM_2.5_ have been described previously [15,16]. Briefly, particles from biomass combustion were obtained from a biomass powerplant (Bürger Energie St. Peter eG, St. Peter, Schwarzwald, Germany), which exclusively combusts chips of soft wood (mainly spruce) from the local forest. The collected bulk fly ash was size-fractionated by a cyclone, and particles of the size fraction with an aerodynamic cut-off diameter of <2.5 µm were used for biological assays. The detailed characterization of the PM_2.5_ fraction is described in our previous studies [15,16]. Lipopolysaccharide content was determined by a limulus amebocyte lysate (LAL) test as described elsewhere [35].

### 2.2. Cell Culture and Treatments

The human bronchial epithelial cell line BEAS-2B was obtained from the American Type Culture Collection (CRL-9609™; Manassas, VA, USA) and maintained in Dulbecco’s Modified Eagle Medium/Ham’s F12 (GE Healthcare, Freiburg, Germany), supplemented with 5 vol% fetal calf serum, 100 IU/mL penicillin and 100 IU/mL streptomycin (all Life Technologies, Carlsbad, CA, USA) at 37 °C in a humidified incubator. Cells were treated with PM_2.5_ for three to five weeks (long-term exposed cells) as previously described [16]. Briefly, cells were passaged twice a week and plated at a density of 7 × 10^5^ cells per 75 cm^2^ cell culture flasks. Following each passage, after one day of recovery (24 h) adherent cells were treated either with 100 μg/mL PM_2.5_ or left untreated for control. After addition of PM_2.5_ cells were maintained in the same medium until the next passage. For biological assays, final exposure experiments were performed in fresh medium without serum, but containing 100 µg/mL PM_2.5_. Controls were supplied with an equivalent volume of medium without PM_2.5_ and serum. The enzyme inhibitors API-2 (TOCRIS Bioscience, Bristol, UK), butylated hydroxyanisole (BHA; Sigma-Aldrich, Steinheim, Germany), L-buthionine-*S*-sulfoximine (BSO; Sigma-Aldrich), IM3829 (Matrix Scientific, Columbia), N-acetylcysteine (NAC; Roth, Karlsruhe, Germany), quinolyl-valyl-O-methylaspartyl-[2,6-difluorophenoxy]-methyl ketone (Q-VD-OPh; MP Biomedicals Europe, Illkirch France), SB203580 (EMD Millipore, Darmstadt, Germany), silibinin (Roth), SP600125 (Abcam, Cambridge, UK), and verapamil (Sigma-Aldrich) were added 1 h before the last exposure to PM_2.5_. Doxorubicin (Sigma-Aldrich) was added 24 h after the last exposure to PM_2.5_. Time of cell harvest is indicated for each experiment.

### 2.3. Viability Assay

Cell numbers were determined by a trypan blue exclusion assay as described previously [36]. The proliferative capacity was evaluated as fold increase of plated cell numbers at the end of each passage. Measurement of cell viability was performed using an [4,5-dimethylthiazol-2-yl]-2,5-diphenyl tetrazolium bromide (MTT) assay. Cells were seeded at a density of 2500 cells per well in a 96-well flat-bottomed cell culture plate and incubated with 0.5 mg/mL MTT (Roth) in phosphate buffered saline for the last 2 h of the experiment. Supernatants were discarded and metabolically reduced formazan crystals were solubilized in 100% dimethyl sulfoxide and quantified at 595 nm using a BIO-RAD iMark^TM^ 96-well microplate absorbance reader (Biorad, Hercules, CA, USA). The relative cell viability was calculated by comparison to the absorbance of the untreated control group (100% viability).

### 2.4. Annexin V Staining

Cells (200,000 cells/well in 6-well plates) were detached by trypsinization, washed in phosphate buffered saline, labeled with FITC Annexin V in 10 mM HEPES/NaOH (pH 7.4), 140 mM NaCl, and 2.5 mM CaCl_2_ (1:100; BD Bioscience, Heidelberg, Germany) for 30 min and analyzed by flow cytometry at an excitation wavelength of 488 nm and an emission wavelength of 520 nm (FL-2; FACS Calibur, BD Bioscience). Data acquisition and analysis were performed using Cell Quest^TM^ Pro 4.0 software (BD Bioscience).

### 2.5. Caspase-3/7—Activity Assay

Cells were seeded in 6-well plates at a density of 100,000 cells/well. After one day of recovery inhibitors were added 1 h prior to PM_2.5_ or medium (control cells) and incubated for 24 h. Subsequently, doxorubicin (final concentration 1 µM) was added for another 24 h before cells were washed with phosphate buffered saline and suspended in lysis buffer (10 mM HEPES-KOH pH 7.9, 0.5 mM ethylene glycol-bis(ß-aminoethy ether)-N,N,N’,N’-tetraacetic acid (EGTA), 0.5 mM ethylenediaminetetraacetic acid (EDTA), 350 µM NaCl, 1 mM MgCl_2_, 1% Nonidet P-40, 20% glycerol, 5 mM dithiothreitol, 2.5 mM phenylmethylsulfonyl fluoride, 14 μg/mL aprotinin). Cell lysis was caused by three freeze–thaw cycles by freezing in liquid nitrogen and thawing in a 40 °C water bath and subsequent vortexing. After centrifugation (13,000× *g*, 4 °C, 10 min) protein concentration was quantified using the Bradford reagent (Quick Start^TM^ Bradford 1 × dye reagent, Bio-Rad, Munich, Germany). The fluorescence emitted by the release of 7-amino-4-methylcoumarin (AMC) from the caspase-3/-7 substrate (Ac-DEVD-AMC, Alexis, Lörrach, Germany) was monitored in a Fluostar Optima plate reader at an excitation wavelength of 370 nm and an emission wavelength of 450 nm. Relative fluorescence unit (RFU) values were calculated via the ratio rate of the fluorescence increase and protein concentration. RFU sample values were referred to control cells and are given as fold increase values.

### 2.6. Intracellular ROS

Intracellular ROS formation was measured using the oxidant sensitive fluorescent probe 2′,7′-dichlorofluorescein diacetate (DCFH-DA; Sigma-Aldrich). Cells were seeded at a density of 70,000 cells/well into 12-well plates, stained with 20 µM DCFH-DA for 30 min and subsequently incubated with 100 µg/mL PM_2.5_ for 6 h to induce ROS generation. After exposure, cells were detached by trypsinization and analyzed by flow cytometry at an excitation wavelength of 485 nm and an emission wavelength of 538 nm. Data acquisition and analysis was performed using Cell Quest^TM^ Pro 4.0 software (BD Bioscience). Sample values were presented as fold increase compared to untreated control cells.

### 2.7. Preparation of Cell Lysates

For whole cell extracts, cells (200,000 cells/well) were lysed in cell lysis buffer (Cell Signaling Technologies, Danvers, MA, USA) according to the manufacturer’s protocol. Nuclear extracts were prepared as described previously [37]. Briefly, cells were suspended in 10 mM HEPES (pH 7.9), 10 mM KCl, 0.1 mM EDTA, 0.1 mM EGTA, 1 mM dithiothreitol, 10 mg/mL leupeptin, 10 mg/mL pepstatin, 20 mg/mL aprotinin, and 1 mM phenylmethylsulfonyl fluoride. After 15 min Nonidet P-40 was added to a final concentration of 0.6%. Samples were centrifuged and the pelleted nuclei were directly lysed in a buffer, containing 20 mM HEPES pH 7.9, 400 mM NaCl, 1 mM EDTA, 1 mM EGTA, 1 mM dithiothreitol, 14 mg/mL aprotinin, 10 mg/mL leupeptin, 10 mg/mL pepstatin, and 1 mM phenylmethylsulfonyl fluoride.

### 2.8. Immunoblotting

Cell lysates were resolved by sodium dodecyl sulfate-polyacrylamide gel electrophoresis (SDS-PAGE) and electrophoretically transferred to a polyvinylidene difluoride membrane (PVDF; EMD Millipore). The membrane was probed with antibodies directed against Akt (#4060; Cell Signaling Technology), phospho-Akt (#9271; Cell Signaling Technology), phospho-JNK (#4668; Cell Signaling Technology), HO-1 (ADI-OSA-110; Enzo Life Sciences, Lausen, Switzerland), Nrf2 (sc-13032; Santa Cruz Biotechnology, Santa Cruz, CA, USA), histone deacetylase-2 (HDAC2; Ab7029; Abcam), or GAPDH (MAB-374; EMD Millipore) according to the specifications of the manufacturers. Specific protein bands were visualized using secondary horseradish–peroxidase-conjugated anti-rabbit or anti-mouse antibodies and enhanced chemiluminescence (NA931, NA934; all GE Healthcare).

### 2.9. Microarray Analysis

The Affymetrix HG-U133 Plus 2.0 oligonucleotide microarrays (Affymetrix, Santa Clara, CA, USA) were used and analyzed as described elsewhere [14,15]. Data have been deposited at https://www.ncbi.nlm.nih.gov/geo/query/acc.cgi?acc=GSE158954.

### 2.10. Measurement of Intracellular Glutathione Levels

Intracellular glutathione (GSH) and the total GSH content (GSH + GSSG) were determined by using the Glutathione Fluorometric Assay Kit according to the manufacturer’s instructions (BioVision, Milpitas, CA, USA). For 48 h, 100,000 cells were seeded into 6-well plates and re-exposed to PM_2.5_ or medium (control cells). The fluorescence was scanned by a 96-well microplate fluorometer (TECAN, Männedorf, Swiss) at an excitation wavelength of 350 nm and an emission wavelength of 420 nm. Sample values were normalized to protein content calculated by using the Pierce^TM^ BCA Protein Assay Kit (Thermo Scientific, Rockford, IL, USA) and expressed as fold increase values compared to untreated control cells.

### 2.11. Cell Morphology

The morphology of BEAS-2B cells was examined by using a light microscope at × 200 magnification (Carl Zeiss Microscopy GmbH, Jena, Germany).

### 2.12. Cellular Doxorubicin Content

Cells (200,000 cells/well in 6-well plates) were treated with 1 µM doxorubicin for 48 h. Subsequently, cells were detached by trypsinization and analyzed by flow cytometry at an excitation wavelength of 488 nm and an emission wavelength of 670 nm (FL3; FACS Calibur, BD Biosciences). Data acquisition and analysis was performed using Cell Quest^TM^ Pro 4.0 software.

### 2.13. Statistical Analysis

Values are shown as mean + standard deviation (SD) for the indicated number of independent experiments. Statistical analysis was performed by the GraphPad PRISM^®^ 5 software (GraphPad Software, San Diego, CA, USA). One and two-way ANOVA were used, and in the case that a significant effect was found, the significance of individual treatments was assessed using Student’s *t*-test and adjusting for multiple comparisons with Bonferroni’s method. Global *p*-values less than 0.05 were considered statistically relevant [* *p* < 0.05; ** *p* < 0.01; *** *p* < 0.001].

## 3. Results

### 3.1. Characterization of PM_2.5_ from Biomass Combustion

PM_2.5_ was obtained from a biomass power plant. The mineralogical composition of the PM_2.5_ fraction was characterized by scanning electron microscopy, X-ray diffraction, and Rietveld refinement and is described elsewhere [16]. PM_2.5_ mainly consisted of a crystalline fraction of inorganic salts (70 wt%) and amorphous components (fly ash spheres, organic material, 30 wt%; Table S1). Heavy metal content could not be quantified, only traces <0.1% were detected. Lipopolysaccharide levels were below the detection limit of the limulus amebocyte lysate (LAL) test (>0.03 EU/mL or 0.3 EU/mg). Polycyclic aromatic hydrocarbon (PAH) analysis was performed previously by GC-APLI-MS and revealed a content of 349.83 µg/kg PM_2.5_, with fluoranthene, pyrene, phenanthrene, and retene presenting the main compounds (Table S2) [15].

### 3.2. Cellular Viability and ROS Generation

**Figure 1:**
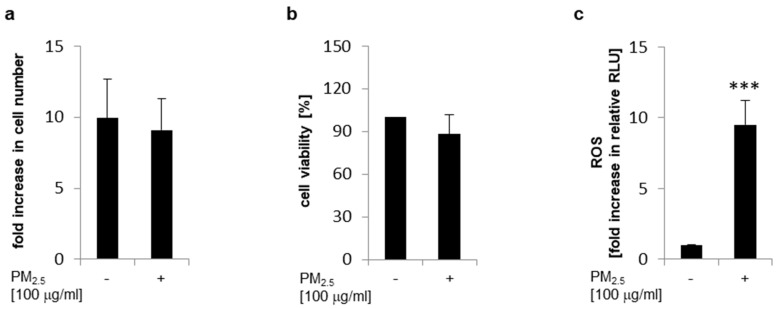
Long-term exposure to PM_2.5_ does not affect proliferation or cellular viability but increases intracellular reactive oxygen species (ROS). BEAS-2B cells were cultured in the presence of 100 µg/mL PM_2.5_ for three to five weeks as described in the materials and methods section. (**a**) Cells were detached by trypsin, analyzed by a trypan blue exclusion assay, and counted in a Neubauer counting chamber using a light microscope. Only viable cells were considered and were depicted as fold increase in cell number compared to plated cells (n = 3). (**b**) Cellular viability was determined by an MTT assay (n = 3). (**c**) For quantification of intracellular ROS, 2′,7′-dichlorofluorescein diacetate (DCFH-DA) labeled cells were analyzed by flow cytometry (n = 3). Cells cultured in the absence of PM_2.5_ served as controls. Values are depicted as means and +standard deviations; *** *p* < 0.001.

Human bronchial epithelial BEAS-2B cells were persistently exposed to PM_2.5_ to mimic the recurrent inhalation of xenobiotic particles present in the ambient air. After three to five weeks no significant difference in cell counts could be observed in the presence or absence of PM_2.5_. Cell numbers were comparable at the end of each passage (Figure 1a). Metabolic activity was also not significantly affected by PM_2.5_ as shown by a cellular viability MTT assay, supporting that PM_2.5_ did not impair the viability of cells (Figure 1b). Furthermore, ROS generation was compared in the presence or absence of PM_2.5_ by labeling cells with the redox sensitive fluorescent probe DCFH-DA and consecutive flow cytometry (Figure 1c). An increase in intracellular ROS could be observed, when cells were persistently exposed to PM_2.5_ from biomass combustion.

### 3.3. Induction of Nrf2

**Figure 2:**
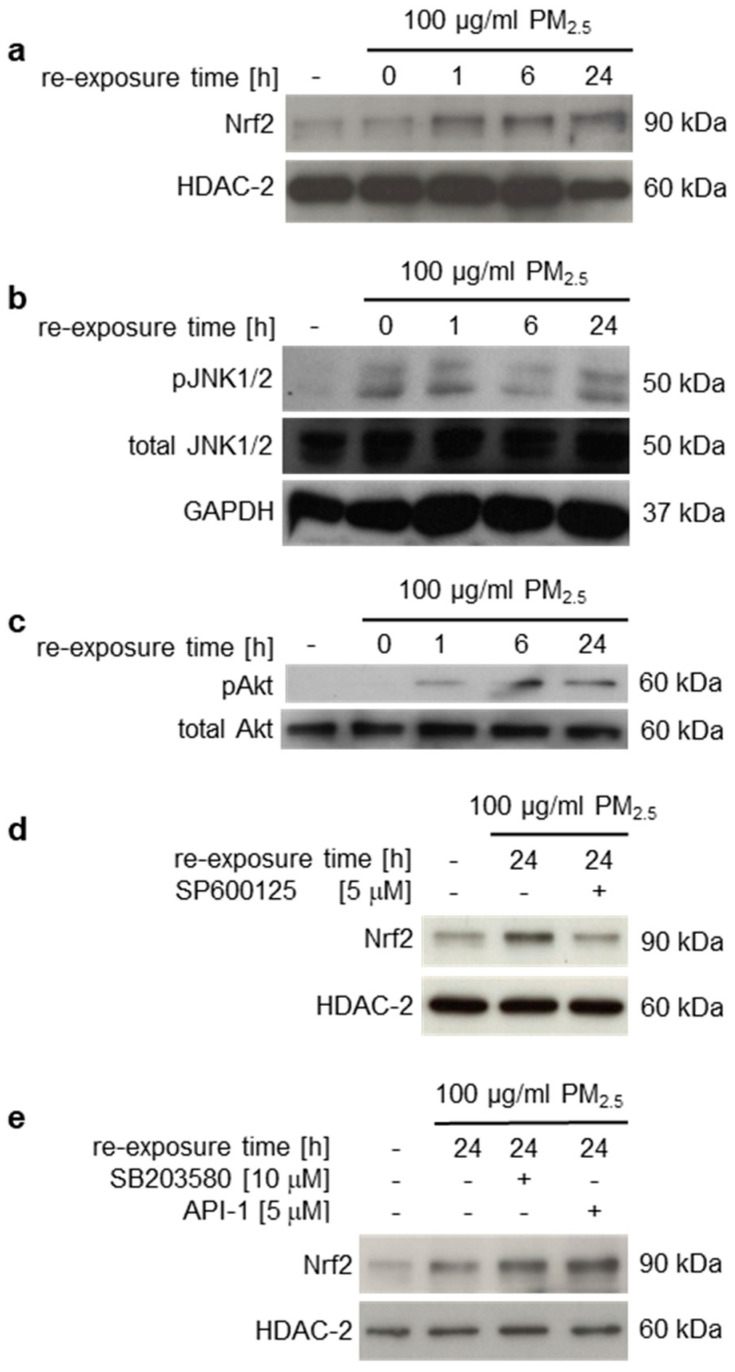
Long-term exposure to PM_2.5_ induces nuclear translocation of Nrf2, which is regulated by JNK1/2. BEAS-2B cells were cultured in the presence of 100 µg/mL PM_2.5_ for 3 to 5 weeks, before final passage and re-exposure of cells to 100 µg/mL PM_2.5_ for the indicated time points (0–24 h). The JNK1/2 inhibitor SP600125 (5 µM), the p38 inhibitor SB203580 (10 µM) or the Akt inhibitor API-2 (5 µM) were added 1 h prior to the last PM_2.5_ re-exposure. (**a**,**d**,**e**) Translocated Nrf2 was detected in nuclear extracts by immunoblotting and normalized to histone deacetylase (HDAC)-2. (**b**,**c**) Phosphorylated JNK1/2 (pJNK1/2) and Akt (pAkt) were immunodetected in non-fractionated cell lysates and normalized to total amounts of JNK1/2, Akt, or GAPDH. Cells, cultured in the absence of PM_2.5_, served as controls (first lane). Inhibition of JNK1/2 decreased nuclear translocation of Nrf2. Representative blots of three different experiments are shown, except of (**e**) (n = 2).

Long-term exposure to PM_2.5_ from biomass combustion did not significantly affect cellular viability (Figure 1a,b) although intracellular ROS levels in PM_2.5_-exposed cells were increased (Figure 1c), indicating an antioxidant stress response. Nrf2 is a master transcriptional regulator of antioxidant genes that have been shown to be important for cytoprotection against ROS and cellular damage [22,23]. Nrf2 is constitutively located in the cytoplasm but it translocates into the nucleus in response to oxidative stress to activate downstream target antioxidant genes [22,23]. To study Nrf2 activation in PM_2.5_-exposed cells, nuclear extracts were prepared and analyzed for translocated nuclear Nrf2. Immunoblots demonstrated a marked increase in nuclear Nrf2 in the presence of PM_2.5_ (Figure 2a). Nuclear translocation of Nrf2 could already be detected at 1 µg/mL PM_2.5_; however, inter-variability of experiments increased at lower concentrations (Figure S1).

The MAPK p38, JNK1/2, or PI3K/Akt pathways may be involved in the activation of Nrf2 [38,39]. Previously, we described a p38-mediated stress response to PM_2.5_ from biomass combustion [16]. Here we additionally show phosphorylation and thus activation of JNK1/2 and Akt by long-term exposure to PM_2.5_ (Figure 2b,c). To clarify whether these kinases affect PM_2.5_-dependent nuclear translocation of Nrf2, specific kinase inhibitors were utilized. Immunoblots of nuclear extracts from PM_2.5_-exposed cells demonstrated that Nrf2 translocation was prevented by the JNK1/2 inhibitor SP600125, but not SB203580 (p38 inhibitor) or API-2 (Akt inhibitor) (Figure 2d,e).

### 3.4. Induction of Nrf2 Downstream Target Genes and Antioxidant Response Prevent Induction of Inflammatory Mediators

**Figure 3:**
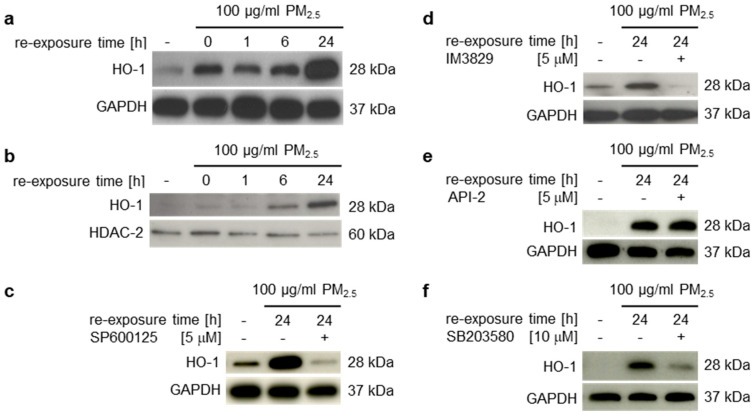
Long-term exposure to PM_2.5_ induces HO-1 expression and nuclear translocation. BEAS-2B cells were cultured in the presence of 100 µg/mL PM_2.5_ for 3 to 5 weeks, before final passage and re-exposure to 100 µg/mL PM_2.5_ for the indicated time points (0–24 h). The JNK inhibitor SP600125 (5 µM), the Nrf2 inhibitor IM3829 (5 µM), the Akt inhibitor API-2 (5 µM), or the p38 inhibitor SB203580 (10 µM) were added 1 h prior to the last PM_2.5_ re-exposure. (**a**,**c**–**f**) Total amounts of HO-1 were detected by immunoblotting in non-fractionated total cell lysates and normalized to GAPDH. (**b**) Translocated HO-1 was immunodetected in nuclear extracts and normalized to HDAC-2. Cells, cultured in the absence of PM_2.5_ served as controls (first lane). Inhibition of JNK1/2 or p38 decreased HO-1 expression. Representative blots of three different experiments are shown, except of (**b**) (n = 2).

HO-1 has emerged as an important phase II detoxifying and anti-oxidative enzyme that is highly upregulated by oxidative stress and is transcriptionally regulated by Nrf2 [24]. To investigate whether nuclear accumulation of Nrf2 is accompanied by an increase of its downstream target genes, HO-1 protein levels were determined in crude total cell lysates or nuclear extracts by immunoblotting (Figure 3a,b). HO-1 was significantly upregulated in human bronchial epithelial BEAS-2B cells in the presence of PM_2.5_, but also when particles were removed for 24 h from long-term exposed cells (Figure 3a, lane 2). Nuclear translocation was most strongly detectable in the presence of PM_2.5_ after 24 h of re-exposure (Figure 3b). Inhibitors for JNK1/2 (SP600125) or Nrf2 (IM3829) prevented HO-1 expression (Figure 3c,d), confirming a JNK1/2-Nrf2-dependent pathway of HO-1 transcriptional activation by PM_2.5_. The Akt inhibitor API-2 had no impact on HO-1 expression, whereas the p38 inhibitor SB203580 partly decreased the PM_2.5_-dependent HO-1 induction (Figure 3e,f). This suggests that an additional mechanism of p38 mediated HO-1 upregulation by fuelwood emissions.

The expression of further Nrf2 target genes was analyzed by using transcriptomic data from Affymetrix Human Genome U133 Plus 2.0 arrays. These arrays have been previously conducted to determine the molecular impact of long-term exposure to identical PM_2.5_ on lung epithelial BEAS-2B cells [14,15]. Functional enrichment using WikiPathway (“WikiPathways”, n.d.) and enriched transcription factor binding sites of upregulated genes after PM_2.5_ exposure identified Nrf2 as a transcription factor involved [15]. Allocated genes included, e.g., *HMOX1* and *SLC7A11* (Table 1). Additionally, further genes controlled by Nrf2 were significantly upregulated and included anti-oxidative or phase II xenobiotic metabolizing enzymes (e.g., *GCLC*, *NQO1*, or *CYP1A1* [28,40,41]; Table 1).

**Figure 4:**
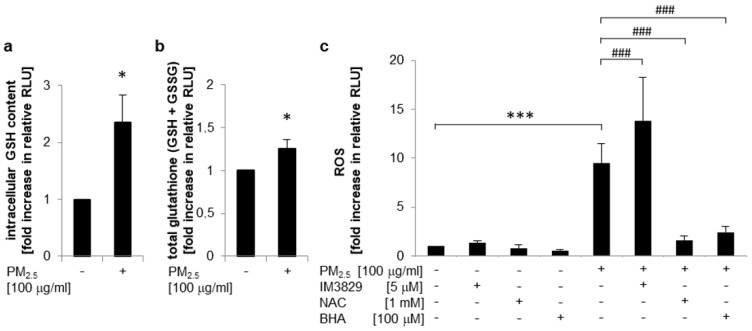
Nrf2 activation by PM_2.5_ increases bioavailability of GSH and limits intracellular ROS levels. BEAS-2B cells were cultured in the presence of 100 µg/mL PM_2.5_ for 3 to 5 weeks, before final passage and re-exposure of cells to 100 µg/mL PM_2.5_. (**a**) Intracellular GSH content or (**b**) increase in total amount of glutathione (GSH + GSSG) after 48 h of re-exposure with PM_2.5_, determined as GSH-associated fluorescence per µg of protein compared to untreated control cells (n = 3). (**c**) Determination of intracellular ROS levels by a DCFH-DA assay using flow cytometry after 6 h of re-exposure with PM_2.5_ (n = 3). The inhibitors BHA (100 µM), IM3829 (5 µM), or NAC (1 mM) were added 1 h prior to PM_2.5_ re-exposure. RLU: relative light units. Values are depicted as means and +standard deviations, compared to untreated control cells: * *p* < 0.05; *** *p* < 0.001; compared to PM_2.5_-exposed cells: ^###^ *p* < 0.001.

Nrf2 plays a pivotal role as a transcriptional regulator of genes encoding for enzymes that are involved in the formation of glutathione [26]. Reduced glutathione (GSH) is the most abundant thiol antioxidant in mammals and a key factor for reducing reactive species, maintaining redox status, and inhibiting apoptosis [26,27]. To analyze whether PM_2.5_-dependent Nrf2 activation is linked to increased intracellular GSH levels, a glutathione fluorometric assay was performed (Figure 4a). Exposure to PM_2.5_ markedly increased both intracellular GSH content (Figure 4a) and total GSH (including GSSG) levels (Figure 4b), indicating that overexpression of *GCLC* (Table 1) by Nrf2 might increase bioavailability of GSH. At the same time, ROS generation by PM_2.5_ was restricted by a Nrf2-mediated pathway and dependent on the cellular anti-oxidative capacity as demonstrated by DCFH-DA labeling of BEAS-2B cells (Figure 4c). In PM_2.5_-exposed cells intracellular ROS generation was significantly increased upon inhibition of Nrf2 by IM3829, but was almost completely blocked in the presence of antioxidant compounds, such as *N*-acetylcysteine (NAC) or butylated hydroxyanisole (BHA). The inhibitors used had no effect on ROS generation in the absence of PM_2.5_. These observations suggest that PM_2.5_-mediated oxidative stress is limited by a Nrf2-dependent mechanism.

At lower oxidative stress levels Nrf2-regulated enzymes exert cytoprotective, anti-oxidative, and anti-inflammatory effects in the lung [20,22,23]. However, escalation of oxidative stress can lead to inflammation and cell death [20]. In our experiments long-term exposure to PM_2.5_ from biomass combustion did not affect cellular viability (Figure 1b). Accordingly, bioinformatic analysis, using the gene data set mentioned above from the Affymetrix Human Genome U133 Plus 2.0 gene expression arrays from PM_2.5_-exposed BEAS-2B cells, did also not reveal any significantly upregulated genes of inflammatory mediators, such as chemokines, cytokines, or matrix metalloproteinases. Interestingly, some genes, such as *CCL2*, *MMP12*, and *MMP13* were even downregulated (Table S3). In conclusion, the Nrf2-mediated response to oxidative stress seems sufficient to prevent PM_2.5_ inflicted inflammatory pathways and cell damage.

### 3.5. Impact of PM_2.5_ on Chemoresistance

**Figure 5:**
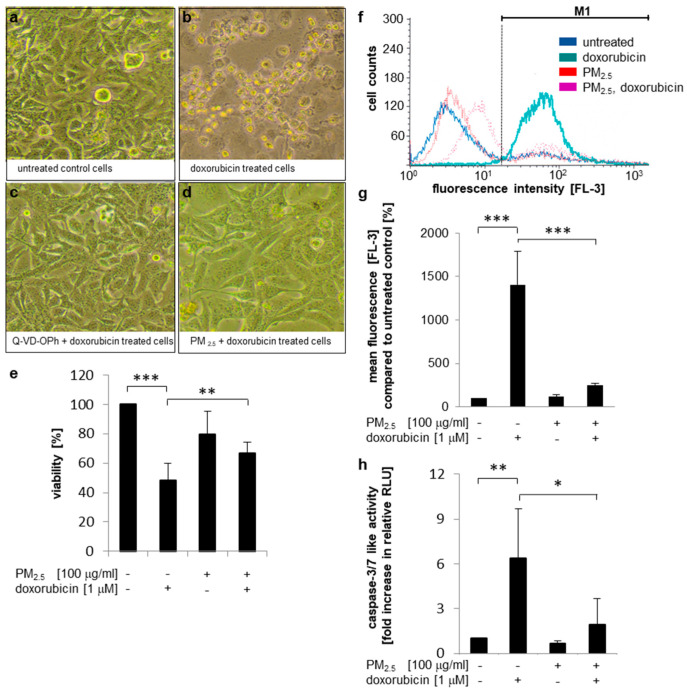
Cell death resistance to doxorubicin mediated by PM_2.5_. BEAS-2B cells were cultured in the presence of 100 µg/mL PM_2.5_ for 3 to 5 weeks, before final passage and re-exposure of cells to 100 µg/mL PM_2.5_ for 48 h. Q-VD-OPh (10 µM) or API-2 (5 µM) were added 1 h prior to the last PM_2.5_ exposure, doxorubicin (1 µM) was added 24 h after the last addition of PM_2.5_. Equally cultured cells that were never exposed to PM_2.5_ served as controls. (**a**–**d**) Photomicrographs of BEAS-2B cells. (**e**) Cell viability determination by an MTT assay (n = 3. (**f**,**g**) Quantification of the intracellular doxorubicin content by flow cytometry. In (**f**), a representative histogram is shown using the FL3 detector, (**g**) depicts mean fluorescence values (FL3) compared to untreated cells (n = 4). M1: gated region for measurement of mean fluorescence. (**h**), detection of apoptosis by a caspase 3/7 activity assay (n = 6). RLU: relative light units. Values are depicted as means and +standard deviations; * *p* < 0.05; ** *p* < 0.01; *** *p* < 0.001.

Hyperactivation of Nrf2 has been correlated with tumor progression and resistance to chemotherapy [26]. Therefore, we investigated whether exposure to PM_2.5_ antagonizes doxorubicin-mediated cell death. Photomicrographs demonstrated that doxorubicin treatment resulted in almost complete rounding and detachment of human bronchial epithelial BEAS-2B cells compared to untreated cells (Figure 5a,b). Cell loss was dependent on the induction of apoptosis, because Q-VD-OPh, a specific pan-caspase inhibitor, prevented cell death (Figure 5c). In contrast, PM_2.5_ pre-exposed cells were not sensitive to doxorubicin-mediated cell death (Figure 5d). In accordance, an MTT assay similarly confirmed that doxorubicin exerts a strong effect on the viability of BEAS-2B cells, which was significantly reduced in PM_2.5_ pre-exposed cells (Figure 5e).

To get further insights in the molecular mechanisms of PM-induced doxorubicin resistance we analyzed whether long-term exposure to PM_2.5_ affects the expression of genes, regulating apoptosis. Gene expression data from the Affymetrix Human Genome U133 Plus 2.0 arrays from PM_2.5_-exposed BEAS-2B cells did not reveal any differentially expressed genes involved in apoptosis (Table S4). Therefore, it can be assumed that PM_2.5_ does not alter the transcriptional balance of pro- or anti-apoptotic mediators, which might have affected susceptibility to cell death.

The intracellular accumulation of doxorubicin was quantified by flow cytometry (Figure 5f,g). Doxorubicin acts as a biocompatible fluorophore [42], which showed highest resolution in the FL3 detector. Doxorubicin treatment for 48 h resulted in a strong accumulation of the fluorophore within control cells that have never been exposed to PM_2.5_. However, in PM_2.5_-exposed cells intracellular doxorubicin content was strongly reduced, suggesting that particle-related resistance is mediated by drug exclusion. Results were correlated to the potential of doxorubicin to induce apoptosis by a caspase-3/7 activity assay (Figure 5h) or by Annexin V binding to the extracellular plasma membrane layer (Figure S2). As expected, caspase activity and Annexin V binding were closely associated with intracellular doxorubicin content. Control cells were highly sensitive to doxorubicin treatment and showed strong caspase-3/7 activation and loss of membrane asymmetry. In contrast, doxorubicin-mediated caspase activation and Annexin V staining were almost completely suppressed in PM_2.5_ exposed cells. Taken together these results suggest that PM_2.5_-exposed cells can exclude chemotherapeutic agents, such as doxorubicin, and that the residual content of doxorubicin is not sufficient to induce apoptosis, thus limiting efficiency of chemotherapy.

**Figure 6:**
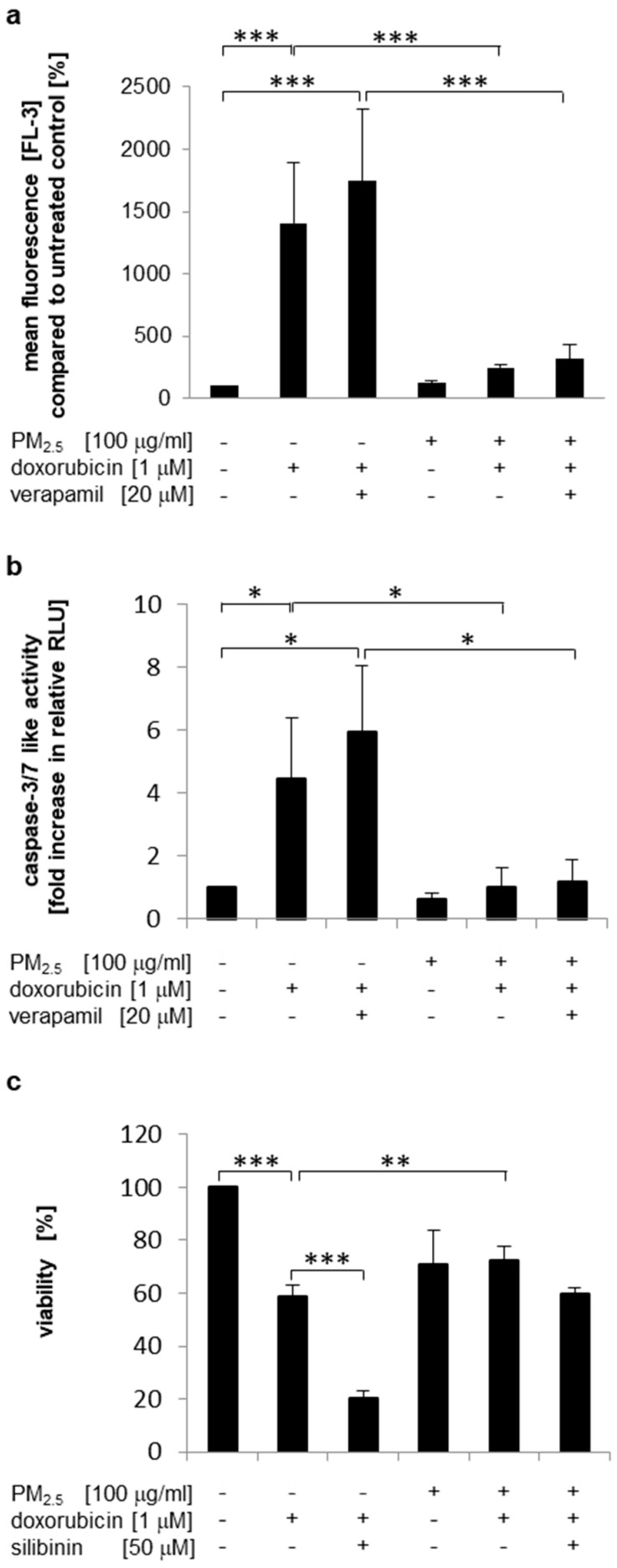
P-gp is not involved in PM_2.5_-mediated doxorubicin resistance. BEAS-2B cells were cultured in the presence of 100 µg/mL PM_2.5_ for 3 to 5 weeks, before final passage and re-exposure of cells to 100 µg/mL PM_2.5_ for 48 h. Verapamil (20 µM) or silibinin (50 µM) were added 1 h prior to the last exposure to PM_2.5_, doxorubicin (1 µM) was added 24 h after the last exposure to PM_2.5_. Cells that were never exposed to PM_2.5_ served as controls. (**a**) Quantification of the intracellular doxorubicin content by flow cytometry. Mean fluorescence values (FL3) were compared to untreated cells (n = 4). (**b**) Detection of apoptosis by a caspase 3/7 activity assay (n = 3). (**c**) Cell viability determination by an MTT assay (n = 3). RLU: relative light units. Values are depicted as means and +standard deviations; * *p* < 0.05; ** *p* < 0.01; *** *p* < 0.001.

Increased efflux of chemotherapeutic agents may be related to the elevated expression of drug efflux transporters [43]. The *ABCB1*-encoded multidrug transporter P-glycoprotein (P-gp/MDR1) has been described as a target for Nrf2 regulation and uses doxorubicin as a substrate [44,45]. However, verapamil, a competitive substrate for P-gp [46], had only a slight, but not significant influence on intracellular doxorubicin levels (Figure 6a and Figure S3a) and the induction of apoptosis (Figure 6b). Additionally, a second P-gp inhibitor (silibinin, [47]) was utilized to analyze doxorubicin resistance in PM_2.5_-exposed cells by a cell viability assay (Figure 6c). Again, cell viability was not significantly reduced by doxorubicin in PM_2.5_-exposed cells (Figure 5e and Figure 6c). Inhibition of P-gp by silibinin only marginally affected the viability of particle-exposed cells (Figure 6c, compare bars 5 and 6), whereas in cells that were never exposed to PM_2.5_, silibinin further enhanced the reduction in cell viability by doxorubicin (Figure 6c, compare bars 2 and 3). These results suggest that P-gp may not be involved in PM_2.5_-mediated doxorubicin resistance.

Next, we analyzed the expression of different drug efflux transporters involved in multiple drug resistance by utilizing the gene expression data from the Affymetrix Human Genome U133 Plus 2.0 arrays from PM_2.5_-exposed BEAS-2B cells. *ABCB1* encoding for P-gp/MDR1 was downregulated, whereas *ABCC2* encoding for the multi resistance-associated protein 2 (MRP2) was upregulated. Further genes for drug efflux transporters involved in multiple drug resistance were not affected (Table S5).

The anti-tumor activity of doxorubicin is critically mediated by the generation of hydrogen peroxide. Consequently, overexpression of antioxidant enzymes can limit doxorubicin sensitivity and apoptosis in tumor cells [48,49,50,51,52]. Therefore, the transcriptomic data from Affymetrix Human Genome U133 Plus 2.0 arrays from PM_2.5_-exposed BEAS-2B cells were also analyzed for differential expression of antioxidant genes involved in resistance to doxorubicin or hydrogen peroxide neutralization (Table S6). However, no upregulation of genes encoding catalase, glutathione peroxidases, peroxiredoxins, superoxide dismutases, or thioredoxins was observed.

**Figure 7:**
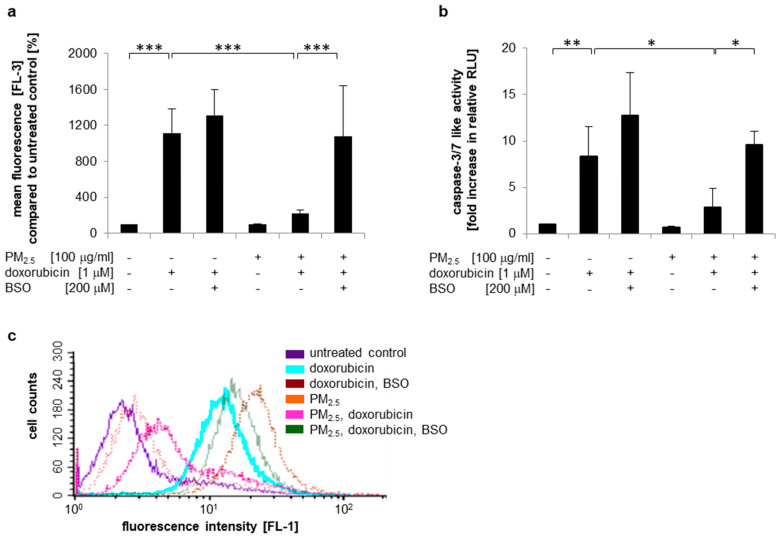
PM_2.5_ reduces doxorubicin resistance by a GSH-dependent mechanism. BEAS-2B cells were cultured in the presence of 100 µg/mL PM_2.5_ for 3 to 5 weeks, before final passage and re-exposure to 100 µg/mL PM_2.5_ for 48 h. BSO (200 µM) was added 1 h prior to the last PM_2.5_ exposure, doxorubicin (1 µM) was added 24 h after the last addition of PM_2.5_. Cells that were never exposed to PM_2.5_ served as controls. (**a**) Quantification of the intracellular doxorubicin content by flow cytometry. Mean fluorescence values (FL3) were compared to untreated cells (n = 3). (**b**) Detection of apoptosis by a caspase-3/7 activity assay (n = 3). (**c**) Detection of apoptosis by Annexin V staining and flow cytometry. A representative histogram is shown (n = 3). RLU: relative light units. Values are depicted as means and +standard deviations; * *p* < 0.05; ** *p* < 0.01; *** *p* < 0.001.

GSH can modulate the efficacy of anti-tumor agents by direct chemical interaction either with the drug or ROS formed as a result of drug activation [26]. We observed both increased intracellular GSH levels (Figure 4a,b) and upregulation of the drug efflux transporter MRP2, which uses GSH-conjugates as a substrate (Table S5). To analyze whether increased bioavailability of GSH is involved in PM_2.5_ induced doxorubicin resistance, intracellular GSH formation was inhibited by BSO, which is known to block glutamate cysteine ligase-dependent GSH synthesis [53]. Subsequently, intracellular accumulation of doxorubicin and the induction of apoptosis were compared in PM_2.5_-exposed cells and controls (Figure 7 and Figure S3b). Similar to our previous observations, cells that were never pre-exposed to PM_2.5_ readily accumulated doxorubicin (Figure 7a and Figure S3b), exhibited strong caspase-3/7 activity (Figure 7b), and displayed loss of membrane asymmetry as visualized by Annexin V binding (Figure 7c). In contrast, PM_2.5_-exposed cells showed a low doxorubicin content and resistance to doxorubicin-mediated apoptosis. Treatment with BSO-abrogated cellular efflux of doxorubicin and increased doxorubicin-mediated caspase activity to levels comparable to those of control cells that were never exposed to PM_2.5_. BSO also prevented the PM_2.5_-mediated stabilization of membrane asymmetry in the presence of doxorubicin. In conclusion, these results indicate that increased bioavailability of GSH is involved in PM_2.5_-mediated chemoresistance.

## 4. Discussion

Here, we demonstrate that long-term treatment with PM_2.5_ from combustion of biofuels induces an anti-oxidative stress response, which includes the activation of Nrf2 and increased bioavailability of GSH. At the same time multiple mediators of cancer initiation and progression were activated. Furthermore, PM_2.5_-exposed cells acquired drug resistance that might complicate therapy of diseases.

Despite locally high PM loads [6,7], direct health effects are rare. They mostly occur after long-term exposure periods and are often limited to especially vulnerable population groups. Accordingly, we did not observe effects on cellular proliferation or cell viability upon prolonged PM_2.5_ exposure. Our findings rather propose that exposure to fine particles may induce a robust molecular defense mechanism that prevents cellular damage and the development of disease, even at high PM concentrations. The Nrf2 pathway, activated by our PM_2.5_ fraction, is known to efficiently restore redox balance upon oxidative stress by transcriptional activation of genes that encode antioxidant and thiol metabolism-associated detoxifying enzymes [23,25]. Accordingly, we determined upregulation of HO-1 and increased bioavailability of GSH, two major anti-oxidative mediators. As a consequence, intracellular ROS levels were decreased in PM_2.5_-treated cells. In this context, studies with the elderly or newborns are interesting, as they indicate that both populations are especially vulnerable to ambient particle exposure [54,55]. An explanation may be an inadequate anti-oxidative response or escalation of oxidative stress in these groups [56,57].

According to the hierarchical stress response model, escalation of oxidative stress can lead to inflammation and finally to cytotoxicity and initiation of programmed cell death [20,21]. Our PM_2.5_ fraction failed to induce an inflammatory response indicating that Nrf2 activation may be sufficient to maintain cellular homeostasis. Relevant upregulated genes encoding for pro-inflammatory cytokines, chemokines, and adhesion molecules were not detected.

Oxidative stress has often been attributed to PAH contaminations in particle emissions [58]. PAHs have also been shown as Nrf2 pathway inductors [59]. Our PM_2.5_ fraction contained most of the PAHs listed by the United States Environmental Protection Agency (EPA), but PAH content was lower than described for other PM sources [15], which might additionally explain an intracellular ROS content not sufficient to induce inflammation or cell death. ROS can directly activate Nrf2 by promoting dissociation from Keap1 [60], but ambient particles can also activate Nrf2 in combination with JNK1/2 and p38 MAPK, or via the PI3K/Akt signaling pathway [61,62]. Accordingly, all of these kinases were activated by PM_2.5_ from biomass combustion. However, only the inhibition of JNK could block nuclear accumulation of Nrf2 and expression of its target gene *HMOX1* encoding HO-1. This indicates that JNK activation is the major pathway of particle induced Nrf2 activation.

The oxidative stress marker DCFH-DA is oxidized by multiple radicals, e.g., hydroxyl radicals, hypochlorous acid, several nitrate and nitrite radicals, superoxide anions, and redox-active metals [63]. Consequently, this study cannot specify the radicals induced by our PM_2.5_ fraction nor it determines the site of their formation. The mitochondrion has been described as a likely source of radical formation upon ambient PM exposure, probably by perturbation of mitochondrial function [64]. Accordingly, we observed a deformation of this organelle in PM_2.5_-treated cells by transmission electron microscopy [16]. Mitochondrial deformation was accompanied by internalization and vesicular accumulation of PM_2.5_ [16]. In fact, endocytosis of particles has been associated with the stimulation of the NADPH oxidase and ROS generation [65]. Consequently, the accumulation, vesicular deposition, and incapability of the digestion and elimination of fine particles might result in permanent cellular stress, the formation of active PAH metabolites, persistent ROS generation, and an anti-oxidative response seeking to neutralize newly generated ROS. Indeed, our PM_2.5_ fraction contains PAHs that were able to induce the PAH-activated aryl hydrocarbon receptor [15]. Additionally, these PAHs have been described to induce the formation of ROS [58]. Whether stimulation of NADPH oxidase or these PAHs are implicated in the described effects remains to be further investigated.

In the present study we observe an anti-oxidative response to PM_2.5_ mainly limited to Nrf2 and the GSH system. Normally, cells are equipped with a repertoire of genes implicated in redox balance regulation [66]. However, in this study superoxide dismutases, glutathione peroxidases, peroxiredoxins, or members of the thioredoxin system were not transcriptionally affected by PM_2.5_. The reason for the observed selective anti-oxidative response is still unclear. Further experiments need to clarify whether the limited anti-oxidative response is correlated to certain radicals, specifically induced by PM_2.5_.

In our study we demonstrated the PM_2.5_-dependent activation of various cytoprotective enzymes and signaling pathways. Activation of Akt may provide cells with a survival signal that allows them to withstand apoptotic stimuli, either by directly targeting pro-apoptotic or anti-apoptotic proteins or by modulating their transcription [29]. Furthermore, we demonstrated upregulation and nuclear accumulation of HO-1, which exhibits anti-oxidative and anti-inflammatory properties and represents an intrinsic defense mechanism to maintain cellular homeostasis and survival [24]. Proteolytically processed HO-1 additionally exerts non-enzymatic functions related to its ability to translocate into the nucleus. In the nucleus it can be responsible for the stabilization and retention of Nrf2, thus prolonging Nrf2 transcriptional activity [67,68]. We also observed nuclear accumulation of Nrf2 in PM_2.5_-exposed cells. Nrf2 performs a critical role in maintaining the cellular redox balance by transcriptional regulation of phase II detoxifying anti-oxidative enzymes, including HO-1 [23,25]. Both the observed Nrf2-dependent HO-1 expression and nuclear retention of Nrf2 by HO-1 might represent a positive feedback loop generating an adaptive response for oxidative stress. Furthermore, the Nrf2-mediated anti-oxidative response increased bioavailability of GSH, the most abundant antioxidant molecule in mammals and a key factor for reducing reactive species or assisting GSH-dependent enzymes that are also involved in the maintenance of the cellular redox state [26]. Additionally, GSH is important for detoxification of endogenous or exogenous compounds, like xenobiotics, by conjugation. In conclusion, induction of Akt, Nrf2, HO-1, and GSH might all represent an effective defense mechanism to encounter oxidative stress and other cell-damaging properties of xenobiotics, which also include anti-cancer drugs.

The induction of pro-survival pathways may also promote the development, progression, and therapy resistance of diseases such as viral infections or cancer. Exposure to PM exacerbates susceptibility to upper respiratory infections [69,70]. Accordingly, activation of Akt has been described to promote viral entry or viral replication and to suppress premature apoptosis, which prolonged virus particle production [71,72]. Similarly, HO-1 can also prolong survival of *Mycobacterium tuberculosis* by activation of the dormancy regulon [73]. PM_2.5_-associated susceptibility to upper respiratory infections may be promoted by such mechanisms.

Previously, we have demonstrated that PM_2.5_ from fuelwood combustion induces epigenetic changes and alters the gene expression profile of persistently exposed human bronchial epithelial cells, which are related to the formation and progression of cancer [73]. Here, we additionally show activation of the Akt pathway and nuclear accumulation of HO-1. Redox signaling via the PI3K/Akt pathway can transform human epithelial cells and results in malignant growth of these cells in nude mice [74]. Furthermore, Akt was shown to be highly expressed in bronchial premalignancies and most non-small cell lung cancers (NSCLC), where it can induce malignant transformation, invasiveness, metastatic growth, and inhibition of programmed cell death [29,75,76]. HO-1 is also known to be highly induced in cancer contributing to carcinogenesis and tumor progression [77]. Nuclear accumulation of HO-1 is typically observed in tumor cells and cancer tissue and is correlated with tumor growth, invasiveness and chemoresistance [77,78].

PM_2.5_ may potentially affect Nrf2-regulated, cancer-relevant pathways. Aberrant activation of Nrf2 has been associated with progression, metastatic invasion and angiogenesis in tumors and is indicative of poor prognosis [26]. Aberrant activation of Nrf2 can lead to deregulation of Nrf2 target genes, including an elevation of intracellular GSH levels [26]. Increased GSH levels have been associated with cellular proliferation and metastasis and thus are particularly relevant in cancer initiation and progression, probably by a combined action with other Nrf2 target genes [26]. We also observed upregulation of *SLC7A11*, a Nrf2 target gene encoding the catalytic subunit xCT of cysteine/glutamate antiporter X_c_^-^, which imports cystine into the cells in exchange for glutamate. Cystine is critical for maintenance of the intracellular GSH level [79]. Overexpression of *SLC7A11* is also associated with a cancer stem-cell-like phenotype and thus may additionally contribute to tumor progression [79]. Nrf2 can also promote tumor angiogenesis by activating HIF1alpha [80]. Previously, we have described activation of HIF1alpha by our PM_2.5_ fraction, indicating a possible cross-talk between these mediators [15]. Some of these mechanisms may be involved in the higher incidence of cancer in PM-exposed individuals.

While ambient air pollutants have extensively been associated with cancer development and progression [81], little is known about survival after cancer diagnosis. Some epidemiologic findings support the hypothesis that air pollution exposure is related to shortened survival [82]. However, the underlying pathophysiological mechanisms are uncertain and may include PM-driven cancer progression after diagnosis or impaired respiratory function [81,83]. Recent data indicate that PAHs may contribute to the development of chemoresistance via the activation of survival signaling and upregulation of anti-apoptotic proteins [84], indicating the need for further studies to highlight air pollution exposures and poor treatment responses.

We observed that PM_2.5_-exposed cells acquire resistance to doxorubicin. Oxygen radical formation is implied as the principal mechanism of doxorubicin-induced apoptotic cell death [48,85]. Accordingly, elevated levels of antioxidant proteins are attributed to increased survival in cancer cells, whereas their transcriptional suppression increases sensitivity to chemotherapy [49,50,51,52]. However, exposure to PM_2.5_ had no impact on the transcription of antioxidant enzymes, including superoxide dismutase 2, glutathione peroxidase 1, peroxiredoxin 1–3,5, or thioredoxin 1, that all have previously been associated with doxorubicin resistance [49,50,51,52]. PM_2.5_ treatment also had no impact on the transcriptional balance of Bcl-2 family members, c-Flip or XIAP. This indicates that PM_2.5_-exposed cells *per se* do not acquire a generally resistant status to cell death by transcriptional deregulation of critical proteins, implicated in the initiation or execution of apoptosis [86,87].

We observed doxorubicin accumulation and induction of apoptosis in control cells, whereas in PM_2.5_-exposed cells intracellular doxorubicin levels and caspase-3/7 activity were reduced and membrane integrity restored, indicating efficient drug neutralization and efflux. Different studies describe doxorubicin resistance and disposition of xenobiotics by P-gp/MDR1 [44,45,88]. Interestingly, here, inhibition of P-gp by verapamil or silibinin did not reverse PM_2.5_-mediated drug resistance, indicating that P-gp is not involved in cellular doxorubicin clearance in PM_2.5_-exposed BEAS-2B cells.

Elevated GSH levels can induce resistance to pro-oxidant chemotherapy, either by forming conjugates with commonly used chemotherapeutic drugs or ROS formed as a result of drug activation and subsequent clearance of the conjugates from the cells [26]. We observed increased intracellular GSH levels, upregulation of the drug efflux transporter *MRP2*, and restoration of doxorubicin-mediated apoptosis upon inhibition of glutamate cysteine ligase-mediated GSH synthesis in PM_2.5_-exposed cells. The latter, in particular, demonstrates that GSH may be critically involved in PM_2.5_-mediated doxorubicin resistance. Recent findings show that tumor cells contain high levels of GSH and that the depletion of GSH by BSO increases the cytotoxicity of doxorubicin [89,90]. In addition, GSH is an important substrate for GSHpx [91]. In the absence of adequate levels of GSH, the GSHpx could not adequately scavenge ROS resulting from the metabolic activation of doxorubicin. Alternatively, thiols might also facilitate the repair of oxidative damage to DNA or other target enzymes [92]. In addition to increased levels of total GSH, we also observed upregulation of *GCLC* and *SLC7A11*. *GCLC* and *SLC7A11* are both involved in GSH synthesis [26] and elevated expression of the *SLC7A11* gene was associated with chemoresistance of cancer cells and considered as a marker for poor patients survival [26]. Because GSH conjugates are formed with multiple conventional chemotherapeutic drugs [26], PM_2.5_-acquired drug resistance is probably not restricted to doxorubicin but may also affect other anti-cancer agents.

Upregulation of the drug efflux transporter *MRP2*, which uses GSH-conjugates as a substrate, might additionally explain attenuation of intracellular doxorubicin accumulation and consequently loss of doxorubicin-mediated apoptosis. Indeed, *MRP2* upregulation is closely associated to chemoresistance [93] and extrusion of doxorubicin [94]. Interestingly, *MRP2* is transcriptionally regulated by Nrf2 [95], thus coupling *MRP2* expression and PM_2.5_-mediated anti-oxidative responses.

Further mechanisms might contribute to PM_2.5_-induced doxorubicin resistance. Akt can be involved in drug resistance by its anti-apoptotic activity. Constitutive expression of Akt is associated with chemoresistance, whereas dominant negative Akt sensitizes cells to anti-cancer drugs [29]. Similarly, HO-1 overexpression is commonly observed in human cancer, where it serves as an essential survival molecule by modulating apoptosis and promoting DNA repair [77]. Chemoresistance is mediated by either nuclear localization of HO-1 or is dependent on HO-1 enzymatic activity [77,78]. Drug metabolizing enzymes like cytochrome P450 (CYP) can also contribute to a resistant phenotype, including doxorubicin (adriamycin). *CYP1A1*, which can be induced via Nrf2 and Ahr [41], was shown to be overexpressed in doxorubicin-resistant cells [96], indicating a possible protective role of cytochrome p450 in doxorubicin-mediated toxicity [97]. In fact, we also observed upregulation of the *CYP1A1* gene in the presence of PM_2.5_. In addition, we have also previously described the induction of autophagy upon long-term exposure to PM_2.5_ in BEAS-2B cells [16]. Autophagy can promote cell survival and chemoresistance [98]. Furthermore, we have identified epigenetic modifications in the promoter of genes encoding microRNA [14], that are related to chemosensitivity and therapy resistance [99,100,101,102,103]. Whether all of these mechanisms participate in cancer drug resistance after PM_2.5_ exposure needs to be clarified by further experiments.

## 5. Conclusions

PM_2.5_ includes a complex mixture of various compounds. Cells probably utilize ancient elimination mechanisms characterized by a broad substrate recognition spectrum for efficient particle neutralization. These may include anti-oxidative enzymes, phase II xenobiotic metabolizing enzymes, and pro-survival pathways. Accordingly, we observed PM_2.5_-mediated induction of HO-1, GSH, upregulation of the *CYP1A1* and *NQO1* genes, and activation of Akt, which are all characterized by responsiveness to multiple targets. Increased bioavailability of GSH due to PM_2.5_ exposure limited oxidative stress and simultaneously mediated acquired cancer-drug resistance to doxorubicin. In conclusion, our observations indicate that the activation of anti-oxidative, detoxifying mediators and survival pathways by PM_2.5_ might be necessary to maintain cellular homeostasis. However, their permanent activation may also promote the development or progression of serious diseases like cancer and negatively influence their treatment.

## Figures and Tables

**Table 1:** Nrf2-associated, upregulated genes upon long-term exposure to PM_2.5._

Gene Symbol	Encoded Protein	*p*-Value	log2FC	FC
*AKR1C1* ^‡^	aldo-keto reductase family 1 member C1	0.0043	1.53	2.89
*AKR1C2* ^‡^	aldo-keto reductase family 1 member C2	0.0038	1.66	3.16
*AKR1C3* ^‡^	aldo-keto reductase family 1 member C3	0.0066	1.25	2.38
*CYP1A1* ^§^	cytochrome P450, family 1, subfamily A, polypeptide 1	0.0011	2.54	5.82
*EGR1* ^†^	early growth response 1	0.0080	1.01	2.02
*EREG* ^‡^	Epiregulin	0.0008	2.73	6.65
*FAM83B* ^‡^	family with sequence similarity 83, member B	0.0058	1.26	2.40
*GCLC* ^§^	glutamate-cysteine ligase, catalytic subunit	0.0197	0.80	1.74
*GOS2* ^‡^	switch protein 2	0.0017	1.25	2.37
*GREM1* ^‡^	gremlin 1, DNA family BMP antagonist	0.0017	2.85	7.20
*HMOX1* ^†,‡^	heme oxygenase (decycling) 1	0.0035	1.23	2.34
*IL1A* ^‡^	interleukin 1, alpha	0.0014	2.32	5.00
*ITGA2* ^‡^	integrin, alpha 2	0.0011	1.54	2.90
*NQO1* ^§^	NAD(P)H dehydrogenase, quinone 1	0.0482	0.71	1.64
*S100A9* ^‡^	S100 calcium binding protein A9	0.0052	2.05	4.15
*SLC7A11* ^†^	solute carrier family 7 (anionic amino acid transporter light chain, xc-system), member 11; xCT	0.0072	1.69	3.24
*SPOCK1* ^‡^	sparc/osteonectin	0.0080	1.04	2.05
*STEAP1* ^‡^	six transmembrane epithelial antigen of the prostate1	0.0059	1.34	2.53
*STC2* ^‡^	stanniocalcin 2	0.0090	0.79	1.73
*TPBG* ^‡^	trophoblast glycoprotein	0.0038	0.93	1.91
*TGFA* ^†,‡^	transforming growth factor, alpha	0.0038	2.04	4.10

^†^ functional enrichment by WikiPathway analysis; ^‡^ according to enriched transcription factor binding sites; ^§^ other Nrf2 associated upregulated genes, relevant for antioxidant or xenobiotic responses.

## Supplementary Materials

Supplementary materials can be found at https://www.mdpi.com/1660-4601/17/21/8193/s1. Table S1: Mineralogical composition of PM_2.5_, Table S2: Concentrations of PAHs (µg/kg) in PM_2.5_ from biomass combustion, Table S3: Expression of cytokine, chemokine, adhesion molecule, and matrix metalloproteinase genes upon long-term exposure to PM_2.5_, Table S4: Expression of genes, relevant for apoptosis after long-term exposure to PM_2.5_, Table S5: Expression of ABC transporter genes, relevant for multidrug resistance after long-term exposure to PM_2.5_, Table S6: Expression of antioxidant redox-sensitive genes upon long-term exposure to PM_2.5_, Figure S1: Long-term exposure to PM_2.5_ induces nuclear translocation of Nrf2, Figure S2: Cell death resistance to doxorubicin is mediated by PM_2.5_, Figure S3: Acquired chemoresistance in PM_2.5_-exposed BEAS-2B cells is mediated by GSH.
