# Supplementary material for: Particulate Matter (PM2.5) from Biomass Combustion Induces an Anti-Oxidative Response and Cancer Drug Resistance in Human Bronchial Epithelial BEAS-2B Cells
Source: Int J Environ Res Public Health. 2020 Nov 6;17(21):8193. doi: 10.3390/ijerph17218193 (PMC7664250; doi:10.3390/ijerph17218193)
Supplement: Supplementary file 1 [file ijerph-17-08193-s001.pdf]

Supporting Information

Particulate matter ($PM_{2.5}$) from biomass combustion induces an anti-oxidative response and cancer drug resistance in human bronchial epithelial BEAS-2B cells

International Journal of Environmental Research and Public Health

Regina Merk[1], Katharina Heßelbach[1], Anastasiya Osipova[1] Désirée Popadić[1], Wolfgang Schmidt-Heck[2], Gwang-Jin Kim[3], Stefan Günther[3], Alfonso García Piñeres[4,5], Irmgard Merfort[1,6,†,‡] and Matjaz Humar[1,†,‡]

[1] Pharmaceutical Biology and Biotechnology, Institute of Pharmaceutical Sciences, Albert-Ludwigs University Freiburg, Freiburg, Germany

[2] Leibniz Institute for Natural Product Research and Infection Biology - Hans-Knöll-Institute (HKI), Jena, Germany

[3] Pharmaceutical Bioinformatics, Institute of Pharmaceutical Sciences, Albert-Ludwigs University Freiburg, Freiburg, Germany

[4] Centro de Investigación en Biología Celular y Molecular (CIBCM), Universidad de Costa Rica, San José, Costa Rica

[5] Escuela de Química, Universidad de Costa Rica, San José, Costa Rica

[6] Spemann Graduate School of Biology and Medicine (SGBM), Albert-Ludwigs University Freiburg, Freiburg, Germany

[†] These authors contributed equally to this work.

[‡] authors for correspondence

Supporting Tables

Table S1. Mineralogical composition of $PM_{2.5}$ †

**Component**	**percentage portion**[b]
Amorphous	30%
Sylvite (KCl)	5%
Syngenite ($K_2Ca(SO_4)_2 \cdot H_2O$	1%
Arcanite (K_2SO_4)	47%
Thenardite (Na_2SO_4)	2%
Merwinite ($Ca_3Mg(SiO_4)_2$)	2%
Larnite (Ca_2SO_4)	5%
Periclase (MgO)	2%
Dolomite ($CaMg(CO_3)_2$)	1%
Portlandite ($Ca(OH)_2$)	2%
Lime (CaO)	1%
Aragonite ($CaCO_3$)	2%

† data depicted from Dornhof et al. [1]; values given in wt%

Table S2. Concentrations of PAHs (μg/kg) in $PM_{2.5}$ from biomass combustion †

PAH	μg/kg	PAH	μg/kg
Acenaphthene	3.64	Naphtho[1,2-*b*]fluoranthene and Naptho[2,3-*a*]pyrene	0.93
Anthracene	7.38	Fluorene	4.67
Benzo[*a*]anthracene	6.70	1-Methylfluorene	1.24
9-Methylanthracene	0.23	7H-Benzo[*c*]fluorene	1.73
9,10-Dimethylanthracene	<0.50	Naphthalene	22.57
6-Methylbenzo[*a*]anthracene	0.84	2-Methylnaphthalene	11.44
5-Methylbenzo[*a*]anthracene	<0.50	2,6-Dimethylnaphthalene	10.63
7,12-Dimethylbenzo[*a*]anthracene	2.27	1,8-Dimethylnaphthalene	0.17
Dibenzo[*a,j*]anthracene	<0.25	Phenanthrene	36.01
Dibenzo[*a,c*]anthracene	<0.25	9-Methylphenanthrene	11.52
Dibenzo[*a,h*]anthracene	<0.25	1-Methylphenanthrene	7.30
Anthanthrene	<2.50	Benzo[*c*]phenanthrene	1.93
3-Methylcholanthrene	<1.25	Pyrene	41.98
Chrysene	15.27	1-Methylpyrene	14.22
2-Methylchrysene	1.31	Benzo[*e*]pyrene	12.27
6-Methylchrysene	0.12	Benzo[*a*]pyrene	5.29
5-Methylchrysene	0.28	6-Methylbenzo[*a*]pyrene	<1.25
4-Methylchrysene	<0.50	Indeno[1,2,3-*cd*]pyrene	3.86
Coronene	2.10	Naphtho[2,3-*e*]pyrene	<0.38
Fluoranthene	50.67	Dibenzo[*a,l*]pyrene	0.49
Benzo[*b*]fluoranthene	12.23	Dibenzo[*a,e*]pyrene	1.72
Benzo[*k*]fluoranthene	2.74	Dibenzo[*a,i*]pyrene	0.38
Benzo[*ghi*]fluoranthene	10.15	Dibenzo[*a,h*]pyrene	<0.50
Naphtho[1,2-*k*]fluoranthene	<0.38	Perylene	0.73
Naphtho[2,3-*b*]fluoranthene	0.59	Benzo[*ghi*]perylene	2.42
Dibenzo[*a,e*]fluoranthene	<0.38	Retene	33.43
Naphtho[2,3-*k*]fluoranthene	<0.38	Triphenylene	6.41

† data depicted from Popadic et al. [2]

Table S3. Expression of cytokine, chemokine, adhesion molecule, and matrix metalloproteinase genes upon long-term exposure to $PM_{2.5}$ †

gene symbol	***encoded protein***	***adjusted p-value***	***log2FC***	***FC***
CCL2	chemokine (C-C motif) ligand 2, monocyte chemotactic protein 1 (MCP1)	0.0043	-1.68	0.31
CCL5	chemokine (C-C motif) ligand 5, RANTES	n.s.	0.01	1.01
CCL8	chemokine (C-C motif) ligand 8, monocyte chemotactic protein 2 (MCP2)	n.s.	-0.06	0.96
CXCL1	chemokine (C-X-C motif) ligand 1	n.s.	0.02	1.01
CXCL10	chemokine (C-X-C motif) ligand 10	n.s.	0.00	1.00
ICAM1	intracellular adhesion molecule 1	n.s.	-0.02	0.98
IL12A	interleukin 12 subunit alpha	n.s.	0.10	1.07
IL12B	interleukin 12 subunit beta	n.s.	-0.01	0.99
MMP1	matrix metallopeptidase 1	n.s.	0.20	1.14
MMP9	matrix metallopeptidase 9	n.s.	-0.02	0.98
MMP12	matrix metallopeptidase 12	0.0009	-2.69	0.16
MMP13	matrix metallopeptidase 13	0.0065	-1.15	0.45
TNF	tumor necrosis factor, TNFalpha	n.s.	-0.05	0.97
VCAM1	vascular cellular adhesion molecule 1	n.s.	-0.04	0.97

† according to Affymetrix Human Genome U133 Plus 2.0 gene expression arrays; n.s., not significant.

Table S4. Expression of genes, relevant for apoptosis after long-term exposure to $PM_{2.5}$ [†]

gene symbol	*encoded protein*	*adjusted p-value*	*log2FC*	*FC*
ATG5 [‡]	autophagy related 5	n.s.	0.02	1.01
BAD, BCL2L8 [§]	Bad, BCL2 associated agonist of cell death	n.s.	0.03	1.02
BAK1, BCL2L7 [§]	Bak, BCL2 antagonist/killer 1	n.s.	0.04	1.03
BAX, BCL2L4 [§]	Bax, Bcl-2-associated X protein	n.s.	0.04	1.01
BCL2 [¶, £]	Bcl-2, B-cell lymphoma 2	n.s.	0.01	0.99
BCL2L1 [¶, £]	Bcl-XL, BCL2 like 1	n.s.	0.07	1.05
BECN1 [‡]	beclin-1, zinc finger protein basonuclin-1	n.s.	-0.01	0.99
BID [§]	Bid, BH3 interacting-domain death agonist	n.s.	0.03	1.01
BIM, BCL2L11 [§]	Bim, Bcl-2-like protein 11	n.s.	0.01	1.01
BIRC5 [¶]	Surviving	n.s.	-0.03	0.98
CFLAR [¶, £]	c-Flip, caspase 8 and FADD-like apoptosis regulator	n.s.	-0.01	0.99
HMGB1 [‡]	high mobility group protein B1	n.s.	0.01	1.01
MCL1 [¶, £]	induced myeloid leukemia cell differentiation protein Mcl1	n.s.	0.03	1.02
XIAP [¶, £]	XIAP, X-linked inhibitor of apoptosis protein	n.s.	0.03	1.03

[†] according to Affymetrix Human Genome U133 Plus 2.0 gene expression arrays; [‡] involved in autophagy, which inhibits apoptosis; [§] promotes apoptosis; [¶] inhibits apoptosis; [£] transcriptional regulation by Akt/NF- B; n.s., not significant.

Table S5. Expression of ABC transporter genes, relevant for multidrug resistance after long-term exposure to $PM_{2.5}$ †

gene symbol	*encoded protein*	*adjusted p-value*	*log2FC*	*FC*
ABCA2	ATP-binding cassette sub-family A member 2	n.s.	-0.01	0.99
ABCB1	P-glycoprotein, P-gp, multi drug resistance protein (MDR)1	0.005	-1.22	0.43
ABCB4	MDR3	n.s.	-0.03	0.98
ABCB11	BSEP (bile salt export pump), sPgp (sister of P-glycoprotein)	n.s.	-0.03	0.98
ABCC1	Multi resistance-associated protein (MRP)1	n.s.	0.03	1.02
ABCC2	Multi resistance-associated protein (MRP)2	0.045	0.50	1.41
ABCC3	Canalicular multispecific organic anion transporter 2, MRP3	n.s.	0.03	1.02
ABCC4	MRP4	n.s.	-0.01	0.99
ABCC5	MRP5	n.s.	-0.03	0.98
ABCC6	MRP6	n.s.	-0.01	1.00
ABCC10	MRP7	n.s.	-0.01	0.99
ABCC11	Multidrug resistance-related protein 8 (MRP8)	n.s.	-0.01	0.99
ABCG2	ATP-binding cassette super-family G member 2	n.s.	0.02	1.01

† according to Affymetrix Human Genome U133 Plus 2.0 gene expression arrays; n.s., not significant.

Table S6. Expression of antioxidant redox-sensitive genes upon long-term exposure to $PM_{2.5}$ [†]

gene symbol	*encoded protein*	*adjusted p-value*	*log2FC*	*FC*
CAT [§]	Catalase	n.s.	0.00	1.00
GPX1 [‡, §]	glutathione peroxidase 1	n.s.	0.01	1.00
GPX2 [§]	glutathione peroxidase 2	n.s.	-0.01	1.00
GPX3 [§]	glutathione peroxidase 3	n.s.	0.02	1.01
GSR	glutathione reductase	n.s.	0.01	1.00
GSS	glutathione synthetase	n.s.	0.03	1.02
PPARGC1A	peroxisome proliferator-activated receptor gamma coactivator 1-alpha	n.s.	-0.07	0.96
PRDX1 [‡, §]	peroxiredoxin 1	n.s.	0.01	1.00
PRDX2 [‡, §]	peroxiredoxin 2	n.s.	-0.04	0.99
PRDX3 [‡, §]	peroxiredoxin 3	n.s.	0.00	1.00
PRDX4 [§]	peroxiredoxin 4	n.s.	0.00	1.00
PRDX5 [‡, §]	peroxiredoxin 5	n.s.	0.01	1.00
PRDX6 [§]	peroxiredoxin 6	n.s.	0.00	1.00
TXN [‡]	thioredoxin 1	n.s.	0.04	1.03
TXN2	thioredoxin 2	n.s.	-0.01	1.00
TXNRD1	thioredoxin reductase 1	n.s.	0.04	1.03
TXNRD2	thioredoxin reductase 2	n.s.	-0.01	0.99
SOD1 [§]	superoxide dismutase 1	n.s.	0.02	1.02
SOD2 [‡, §]	superoxide dismutase 2	n.s.	0.00	1.00
SOD3 [§]	superoxide dismutase 3	n.s.	0.00	1.00

† according to Affymetrix Human Genome U133 Plus 2.0 gene expression arrays; ‡ involved in doxorubicin resistance; § involved in H_2O_2 generation/detoxification; n.s., not significant.

Supporting Figures

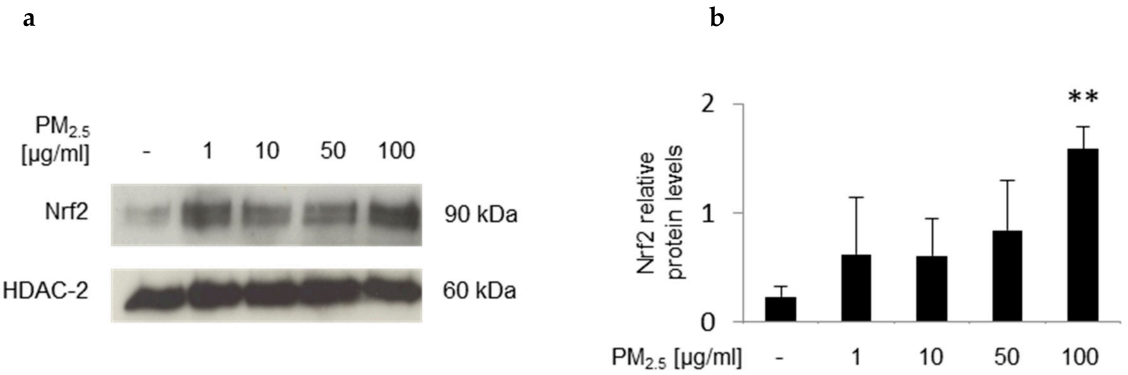

Figure S1. Long-term exposure to $PM_{2.5}$ induces nuclear translocation of Nrf2. BEAS-2B cells were exposed to 1 - 100 g/ml $PM_{2.5}$ for 12 h. (**a**), translocated Nrf2 was detected in nuclear extracts by immunoblotting and normalized to histone deacetylase (HDAC)-2. Cells, cultured in the absence of $PM_{2.5}$ served as controls (first lane). A representative blot of two different experiments is shown. (**b**), quantification of immunoblots relative to control. Statistical analysis was performed by using the R statistical software [3] and determined by analysis of variance (ANOVA), followed by Dunnett's post-hoc test. Values are depicted as means and +standard deviations; **, $p<0.01$.

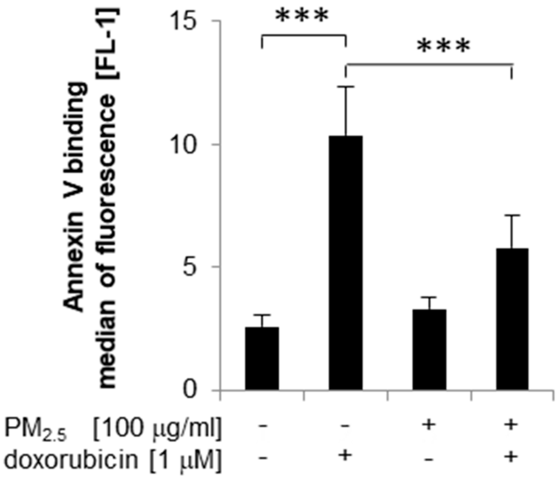

Figure S2. Cell death resistance to doxorubicin is mediated by $PM_{2.5}$. BEAS-2B cells were cultured in the presence of 100 µg/ml $PM_{2.5}$ for 3 to 5 weeks, before final passage and re-exposure of cells to 100 µg/ml $PM_{2.5}$ for 48 h. Doxorubicin (1 µM) was added 24 h after the last addition of $PM_{2.5}$. Equally cultured cells that were never exposed to $PM_{2.5}$ served as controls. Detection of apoptosis by Annexin V staining and flow cytometry (n = 4). Statistical analysis was performed by using the R statistical software [3] and determined by analysis of variance (ANOVA), followed by the Tukey's post-hoc test. Values are depicted as means and +standard deviations; **$p<0.01$; ***$p<0.001$.

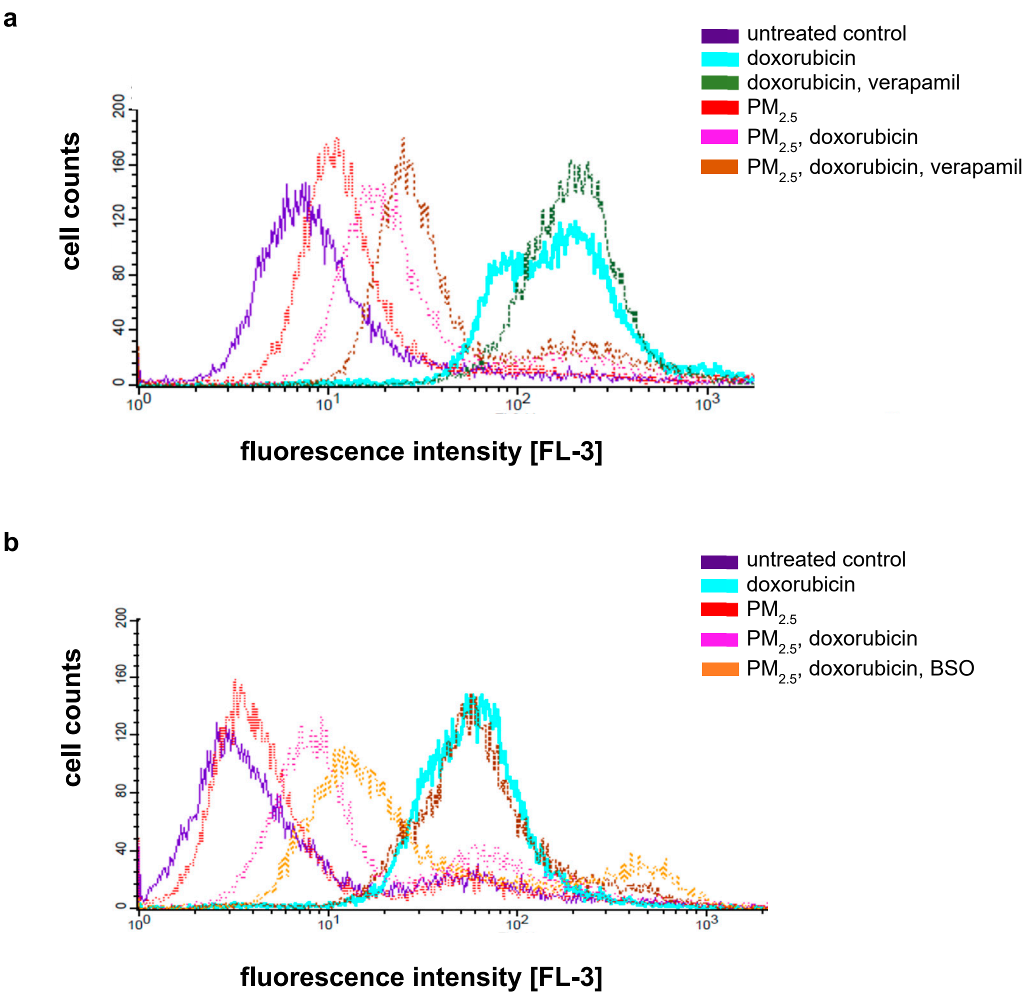

Figure S3. Acquired chemoresistance in $PM_{2.5}$ exposed BEAS-2B cells is mediated by GSH. BEAS-2B cells were cultured in the presence of 100 g/ml $PM_{2.5}$ for 3 to 5 weeks. After final passage, cells were re-exposed to 100 g/ml $PM_{2.5}$ after 24 h. Verapamil (20 M) or BSO (200 M) were added 1 h prior to the last $PM_{2.5}$ exposure. Doxorubicin (1 M) was added 24 h after the last $PM_{2.5}$ exposure for an additional 48 h. Cells that were never exposed to $PM_{2.5}$ served as controls. Intracellular doxorubicin content was determined by flow cytometry and is depicted as histograms. (**a**), inhibition of P-gp/MDR1 by verapamil; (**b**), inhibition of GSH synthesis by BSO.

References

1. Dornhof R, Maschowski C, Osipova A, Giere R, Seidl M, Merfort I, et al. Stress fibers, autophagy and necrosis by persistent exposure to PM2.5 from biomass combustion. PLoS One. 2017;12(7):e0180291. doi: 10.1371/journal.pone.0180291. PubMed PMID: 28671960; PubMed Central PMCID: PMCPMC5495337.
2. Popadic D, Hesselbach K, Richter-Brockmann S, Kim GJ, Flemming S, Schmidt-Heck W, et al. Gene expression profiling of human bronchial epithelial cells exposed to fine particulate matter (PM2.5) from biomass combustion. Toxicol Appl Pharmacol. 2018;347:10-22. doi: 10.1016/j.taap.2018.03.024. PubMed PMID: 29596927.
3. R Core Team (2019). R: A language and environment for statistical computing. R Foundation for Statistical Computing, Vienna, Austria. URL https://www.R-project.org/
